# Echinococcosis Risk among Domestic Definitive Hosts, Japan

**DOI:** 10.3201/eid1302.051377

**Published:** 2007-02

**Authors:** Masao Kamiya, Jose Trinipil Lagapa, Sumiya Gansorig, Fumiyo Kobayashi, Nariaki Nonaka, Yuzaburo Oku

**Affiliations:** * Rakuno Gakuen University, Hokkaido, Japan; † Central Mindanao University, Musuan, the Philippines; ‡ Hokkaido University, Hokkaido, Japan; § Forum on Environment and Animals, Hokkaido, Japan

**To the Editor:** Echinococcosis is a serious parasitic zoonosis in the Northern Hemisphere. In Japan, it is characterized by alveolar, hepatic, and cerebral disorders in humans caused by the larval form (metacestode) of the tapeworm *Echinococcus multilocularis*. The life cycle of the parasite is maintained in the wild by gray-backed voles, *Clethrionomys rufocanus*, as intermediate hosts, and by red foxes, *Vulpes vulpes*, as definitive hosts. Humans are infected by ingestion of the parasite eggs, mainly through water contaminated with the feces of wild red foxes, which have an estimated infection prevalence of 54%–56% (*1*).

The echinococcosis-endemic area in Japan is restricted to the northern island of Hokkaido, although sporadic human cases have been reported on other islands (*2*), and infected pigs have been documented on the main island of Honshu (*3*). While the threat of echinococcosis spreading into Honshu had raised fears, an emergent concern is the possible role of domestic dogs in dispersing the disease from disease-endemic areas during relocation of residences by owners or when accompanying owners during domestic travel.

In September 2005, a stray dog in Saitama prefecture in mainland Honshu was found to be positive for *E. multilocularis* infection by PCR (mitochondria 12S RNA gene) (Y. Morishima, pers. comm.). The sequence was identical to the Hokkaido isolate (GenBank accession no. AB244598). This raised an alarm because the area in which the infection was found is adjacent to the Tokyo metropolis, the most populous zone in Japan. Reports also claimed that 2 of 69 dogs moved from Hokkaido to Honshu were positive for *E. multilocularis* by coproantigen examination (*4*).

Nearly 10,000 pet dogs were estimated to have been transported in 1 year to and from Honshu and Hokkaido by planes and ferries; this presumably included up to 30 *E. multilocularis*–infected pet dogs per year (*5*). Even so, no compulsory quarantine or *Echinococcus* examination is enforced for dogs transported within Japan. A compulsory requirement of a certificate from a veterinarian stating that the animal has been treated with praziquantel 3–4 days before traveling would be a helpful preventive measure. As part of an amendment to the Infectious Disease Law in Japan, *E. multilocularis* infection was included among the 4th Category Diseases (*6*). Thus, since October 2004, it has been mandatory for veterinarians who have diagnosed echinococcosis in dogs to report each case to health authorities, the first national reporting system of its kind worldwide.

Our laboratory established the Forum on Environment and Animals (FEA) to meet the demand for accurate and rapid diagnosis of echinococcosis in domestic dogs. FEA is a hub for veterinary practitioners around the country for confirmation of *E. multilocularis* infection in definitive hosts, especially dogs but also cats. Feces submitted are from dogs and cats that are suspected to be infected and that wander or walk in parks and woodlands and likely prey on wild rodents. Examinations are performed weekly, and results are immediately forwarded to the submitting veterinarians. Before examination, fecal samples are sterilized by heating for 12 hours at 70°C. Fecal egg examination is conducted by using centrifugal flotation (*7*) with sucrose solution with a specific gravity of 1.27. Sandwich ELISA using a monoclonal antibody EmA9 (*8*) is used for *E. multilocularis* coproantigen detection. Egg- and ELISA-positive fecal samples from dogs are subjected to PCR amplification (mitochondria 12S RNA gene) (*9*).

The Table presents data of samples from both dogs and cats examined by FEA from April 2004 through August 2005. A total of 1,460 domestic dogs were examined, and 4 (0.27%) were confirmed positive to echinococcosis by PCR, all from Hokkaido. Test results from eggs detected in cat feces suggested these animals were infected with *Taenia taeniaeformis*, a cat tapeworm, rather than *E. multilocularis*, because coproantigen ELISA results were negative and an ELISA-positive sample did not contain eggs.

**Table:** Prevalence of echinococcosis in definitive hosts subjected to fecal egg examination, ELISA coproantigen test, and PCR copro-DNA detection, Japan

Species	No. samples	Positive samples (%)		
Egg examination	ELISA	PCR
Dogs	1,460	3 (0.20)	6 (0.41)	4 (0.27)
Cats	128	4 (3.12)	1 (0.78)	ND*
Total	1,588	7 (0.44)	7 (0.44)	–

*ND, not done.

To our knowledge, this survey registered the greatest number of domestic dogs examined recently in Japan for echinococcosis. Confirmed cases of infection in dogs further showed the potential threat of domestic dogs transmitting *E. multilocularis* to humans in this region, as well as the potential for dispersal to other islands of Japan if proper preventive measures are not implemented.

A previous report of necropsy examinations of 9,849 dogs from 1966 to 1999 showed a prevalence of 1.0% (*10*). Although necropsy is considered the most reliable method to diagnose *E. multilocularis* in definitive hosts, it is not applicable for live animals such as domestic dogs and cats. Fecal egg examination is generally used; however, infection is difficult to confirm because the morphology of taeniid eggs is indistinguishable from those of *E. multilocularis*, and eggs are excreted intermittently even after the worms mature. Coproantigen detection had proven useful for primary screening and was documented to have 94.9% sensitivity and 100% specificity for echinococcosis in wild red foxes in Hokkaido (*1*). The combined egg examination, ELISA, and PCR methods we used showed an accurate and rapid diagnosis in domestic dogs, which is important for immediate reporting, treatment, and action to safeguard dog owners.
